# Laundry detergent promotes allergic skin inflammation and esophageal eosinophilia in mice

**DOI:** 10.1371/journal.pone.0268651

**Published:** 2022-06-27

**Authors:** Jamie Tanzer, Di Meng, Asa Ohsaki, Julie M. Caldwell, Melissa K. Mingler, Marc E. Rothenberg, Michiko K. Oyoshi

**Affiliations:** 1 Harvard College, Cambridge, MA, United States of America; 2 Division of Pediatric Allergy, Mucosal Immunology and Biology Research Center, Massachusetts General Hospital for Children, Charlestown, MA, United States of America; 3 Department of Pediatrics, Harvard Medical School, Boston, MA, United States of America; 4 Division of Immunology, Boston Children’s Hospital, Boston, MA, United States of America; 5 Division of Allergy and Immunology, Department of Pediatrics, Cincinnati Children’s Hospital, University of Cincinnati College of Medicine, Cincinnati, OH, United States of America; University of Maryland School of Medicine, UNITED STATES

## Abstract

The prevalence of allergic diseases is on the rise, yet the environmental factors that contribute to this increase are still being elucidated. Laundry detergent (LD) that contains cytotoxic ingredients including microbial enzymes continuously comes into contact with the skin starting in infancy. An impaired skin barrier has been suggested as a route of allergic sensitization. We hypothesized that exposure of skin to LD damages the skin barrier resulting in systemic sensitization to allergens that enter through the impaired skin barrier. Mouse skin samples exposed *in vitro* to microbial proteases or LD exhibited physical damage, which was more pronounced in neonatal skin as compared to adult skin. Exposure of the skin to microbial proteases *in vitro* resulted in an increase in the levels of interleukin (IL)-33 and thymic stromal lymphopoietin (TSLP). BALB/c wild type mice epicutaneously exposed to LD and ovalbumin (OVA) showed an increase in levels of transepidermal water loss, serum OVA-specific immunoglobulin (Ig) G1 and IgE antibodies, and a local increase of *Il33*, *Tslp*, *Il4* and *Il13* compared with LD or OVA alone. Following intranasal challenge with OVA, mice epicutaneously exposed to LD showed an increase in allergen-induced esophageal eosinophilia compared with LD or OVA alone. Collectively, these results suggest that LD may be an important factor that impairs the skin barrier and leads to allergen sensitization in early life, and therefore may have a role in the increase in allergic disease.

## Introduction

The prevalence of allergic diseases has been increasing in the last few decades [1–6]. A combination of genetic factors and changes in the environment such as microbiota and lifestyle in industrialized countries, have been suggested as a cause of the increased prevalence of allergies, however, the environmental factors that have a key role in this increase remain largely unknown.

The skin barrier protects against environmental insults that include physical stresses, chemical exposure, microbial assaults, and allergens [7–11]. Skin barrier dysfunction is a hallmark of atopic dermatitis (AD) [12], a chronic pruritic inflammatory skin disease that affects ~15% of children and 1–3% of adults in the U.S. [13,14]. An impaired skin barrier in AD has been suggested as the initial entry point for food allergens resulting in food allergy [15]. In addition, eosinophilic esophagitis (EoE), a chronic inflammatory allergic disorder with increasing prevalence, is characterized by the accumulation of eosinophils in the esophagus [16], and patients with EoE frequently have concurrent AD or a history of AD [17]. Both diseases are associated with allergen-specific T helper (Th) 2 responses, production of allergen-specific immunoglobulin (Ig), local eosinophilia, and local impaired barrier function [18–20]. The observation that childhood AD can predispose to the progressive development of allergic diseases, suggests that the disrupted skin barrier in AD could be a potential risk factor for the development of subsequent food allergy and EoE [21–23].

The frequencies of allergic diseases, such as food allergies and AD, are higher in infants and children than in adults [4,24]. Neonatal skin physiology differs dramatically from adult skin, and children are more vulnerable to environmental insults until at least two years of age [25]. Among the many environmental factors associated with the modern lifestyle and the rise in allergic diseases, laundry detergent (LD) is particularly relevant to the skin barrier, especially in patients with AD [26,27]. Microbial proteases were first introduced to LD in 1960’s [28], then replaced with proteases from genetically modified bacteria that are more resistant to changes by temperatures and pH, hence could act at body temperatures and survive laundry dryer machine cycles [29–31]. Proteases in LD were soon recognized as a serious occupational health hazard for production workers due to their ability to cause skin irritation, respiratory symptoms, asthma, and allergy to proteases and common allergens [32–35]. Consumers also developed similar allergic symptoms associated with the dose of LD [36–42], which highlighted the risk of low-level but long-term exposure to LD and their residues through inhalation or skin exposure to LD or their residues on clothing [43]. In 1992, the US Environmental Protection Agency and Department of Energy created the ENERGY STAR program that limited the usage of water by washing machines due to concerns for the environment [44,45]. Accordingly, LD manufacturers developed high-efficiency LD to improve rinsing in reduced water volumes [46], which resulted in significant levels of LD residues remaining on cloths [47]. The activity of LD residues on cloths can be sustained by the lower temperature of the environment-friendly washer and dryer, the enhanced stability of proteases from genetically modified bacteria, and the higher moisture levels on the infant’s skin. These key changes made to LD in recent decades may contribute to the escalation in allergies.

In this study, we hypothesized that skin exposure to protease-containing LD or LD-associated proteases impairs the skin barrier, which results in systemic allergen sensitization and subsequent allergic inflammation. We demonstrate that mouse samples of adult and neonatal skin exposed *in vitro* to microbial proteases or LD, exhibited physical damage that was more prominent in neonatal skin as compared to adult skin. Exposure of skin to microbial proteases *in vitro* increased the levels of interleukin (IL)-33 and thymic stromal lymphopoietin (TSLP), which drive type 2 immunity [48,49]. BALB/c wild type (WT) mice epicutaneously exposed to LD and ovalbumin (OVA) developed allergic skin inflammation, as indicated by increased transepidermal water loss (TEWL), dermal eosinophil infiltration, and a local increase of *Il33*, *Tslp*, *Il4* and *Il13*. Mice exposed to LD and OVA exhibited increased serum OVA-specific IgG1 and IgE antibodies and Th2 cytokine secretion by splenocytes compared with LD or OVA alone. In addition, mice exposed to LD and OVA showed an increase in allergen-induced esophageal eosinophilia following intranasal challenge with OVA compared with LD or OVA alone. Collectively, these results suggest that exposure to LD disrupts the skin barrier and leads to systemic sensitization and development of subsequent allergy. As such, LD exposure in early life may be a key environmental factor that plays a role in the increase in allergic disease.

## Materials and methods

### Mice

BALB/c WT mice were purchased from Taconic. All mice were bred in the animal facility of BCH, and kept in a specific pathogen-free environment and fed OVA-free diet. All animal studies were performed according to the protocols reviewed and approved by the Institutional Animal Care and Use Committee at Boston Children’s Hospital, Cincinnati Children’s Hospital Medical Center and the Center for Comparative Medicine at Massachusetts General Hospital. All potential painful procedures were done under the guidelines for aseptic rodent surgery with isoflurane titrated to provide anesthesia. Adequacy of dosage was assessed by response to foot pad pressure, pattern of respiration, and lack of movement during the procedures. Carbon dioxide inhalation was used for euthanasia.

### Allergen sensitization and challenge

The dorsal skin of anesthetized two- to three-week-old BALB/c mice was shaved and rested for two days for recovery from any injury by shaving [50,51]. Ten percent Tide Original Scent Liquid Laundry Detergent (P&G) as LD in 100 μl of normal saline was incubated at 60 ˚C for 10 minutes to un-encapsulate proteases to better represent the real-life use of LD in washing machine. Tide was used since it is the most common brand used in the U. S. by a wide margin [52]. LD or placebo (100 μl of normal saline), was placed on a patch of sterile gauze (1x1 cm), which was secured to the dorsal skin with Tegaderm to minimize oral consumption of LD and kept for 6 hours. After removal of the gauze containing LD, the area was immediately covered with a patch of sterile gauze with 100 μg OVA (Grade V; Sigma) in 100 μl of normal saline and secured to the dorsal skin with Tegaderm to minimize oral exposure to allergen and kept for 18 hours. Mice received two cycles of daily exposure to LD and OVA for 5 consecutive days with an interval of 2 days (see **Fig 2A**). TEWL was measured by Tewameter TM300, which measures the density gradient of the water evaporation from the skin, 24 hours before and after application of LD. The probe was held in place on the skin for one TEWL measurement of 40 seconds. For some experiments, 24 hours after the last sensitization, anesthetized mice were intranasally challenged with 3 mg OVA in 25 μl of normal saline per nostril every other day for 5 days (see **Fig 3A**). Mice were euthanized 24 hours after the last sensitization or intranasal challenge to harvest tissues.

### Skin biopsy culture

Five-mm^2^ skin biopsies were collected from the dorsal skin of 8-week-old adult and 9-day-old neonatal BALB/c mice. Skin samples were incubated for 24 hours at 37° C in DMEM supplemented with 5% fetal calf serum and 1% penicillin streptomycin (Gibco) containing 0.00002% Alcalase, Esperase, or Savinase (all from Sigma) that are commonly used across multiple LDs or Tide as identified from patent filings, including P&G filing (an owner of Tide) [53]. Since the exact levels of enzymes in LD such as Tide are proprietary, we calculated the dose of proteases as physiologically accurate as possible in our *in vitro* experiment. The concentrations of proteases were selected based on Human & Environmental Risk Assessment (HERA) estimates of protease exposure to skin from fabric wear [54]. The levels of protease residues on fabric were estimated at 0.35 μg/g after various washing conditions with commercial LD products, fabric compositions, and number of washing cycles [54]. The concentrations of proteases in contact with the skin were calculated as the followings: 0.35 μg/g (protease residues on fabric) x 0.01 μg/cm^2^ (fabric density)/0.01 cm (skin film thickness) = 0.35 μg/cm^3^ = 0.35 μg/ml = 3.5 x 10^−7^ g/ml = 3.5 x 10^−5^% (w/v) = 0.00003% (w/v). The concentrations of LD *in vitro* were selected to contain similar percentages of proteases based on the range of protease concentrations in commercial LD products (0.2%-2%) [54]. For cytokine measurements, supernatants were collected 1 hour after stimulation with Savinase and analyzed with ELISA kits according to manufacturers’ protocols (eBioscience, Biolegend).

### Histological analysis

Multiple 4 μm sections of mid-esophagus were stained with hematoxylin and eosin (H&E) or with antiserum against eosinophil major basic protein (MBP) as previously described [55]. Slides were photographed at 10x and 20x, blinded, assigned a unique number to our study’s samples, and scored comparatively based on physical damage, taking into account epidermal thickening and ridging (protective mechanism), spongiosis, separation of keratin, epidermal, and dermal layers, and degradation of adnexal matrix and muscle tissue (see examples in **S1 Fig**). An unblinded control sample was used as the comparator for analysis of the other samples. Eosinophils were counted in a blinded fashion in 5–10 different randomly chosen high-power fields (HPFs) at a magnification of 400X.

### Flow cytometry

These assays were performed as described previously [56]. Briefly, Esophageal tissue of mice was opened longitudinally, digested in 1 mg/ml collagenase IV (Worthington Biochemical) and 20 μg/ml DNase (Sigma) for 30 minutes, and mashed through 70 μm nylon mesh filters. Single-cell suspensions were incubated with Fixable Viability Dye (eBioscience) for dead cell exclusion and stained with fluorochrome-conjugated mAbs for Siglec-F, IgE, c-kit, CD3, CD11c, CD19, CD45, CD49b, NKp46 (purchased from Biolegend, BD Biosciences, or eBioscience). Mouse eosinophils were identified as live, CD45^+^lin (CD3, CD19, CD11c, NKp46)^-^Siglec-F^+^ side-scatter (SSC)^high^ cells. Mouse basophils were identified as live, CD45^+^lin^-^c-kit^-^CD49b^+^IgE^+^ cells.

### Quantitative PCR analysis

Total RNA was extracted from homogenized skin tissue and quantitative real-time PCR was performed as described previously [57].

### Serum antibody determination and *in vitro* cytokine production

These assays were performed as described previously [57,58].

### Statistical analysis

A nonparametric Mann-Whitney’s U test was used to compare the distribution of each outcome between two groups. Nonparametric one-way ANOVA with Dunn’s multiple comparison tests were used to compare three or more groups. Two-way ANOVA with Bonferroni multiple comparisons were used to determine two independent variables. All analyses were performed using the Graphpad Prism version 5.0 (Graphpad Software).

## Results

### LD or microbial proteases cause skin damage in mouse skin *in vitro*

**Fig 1:**
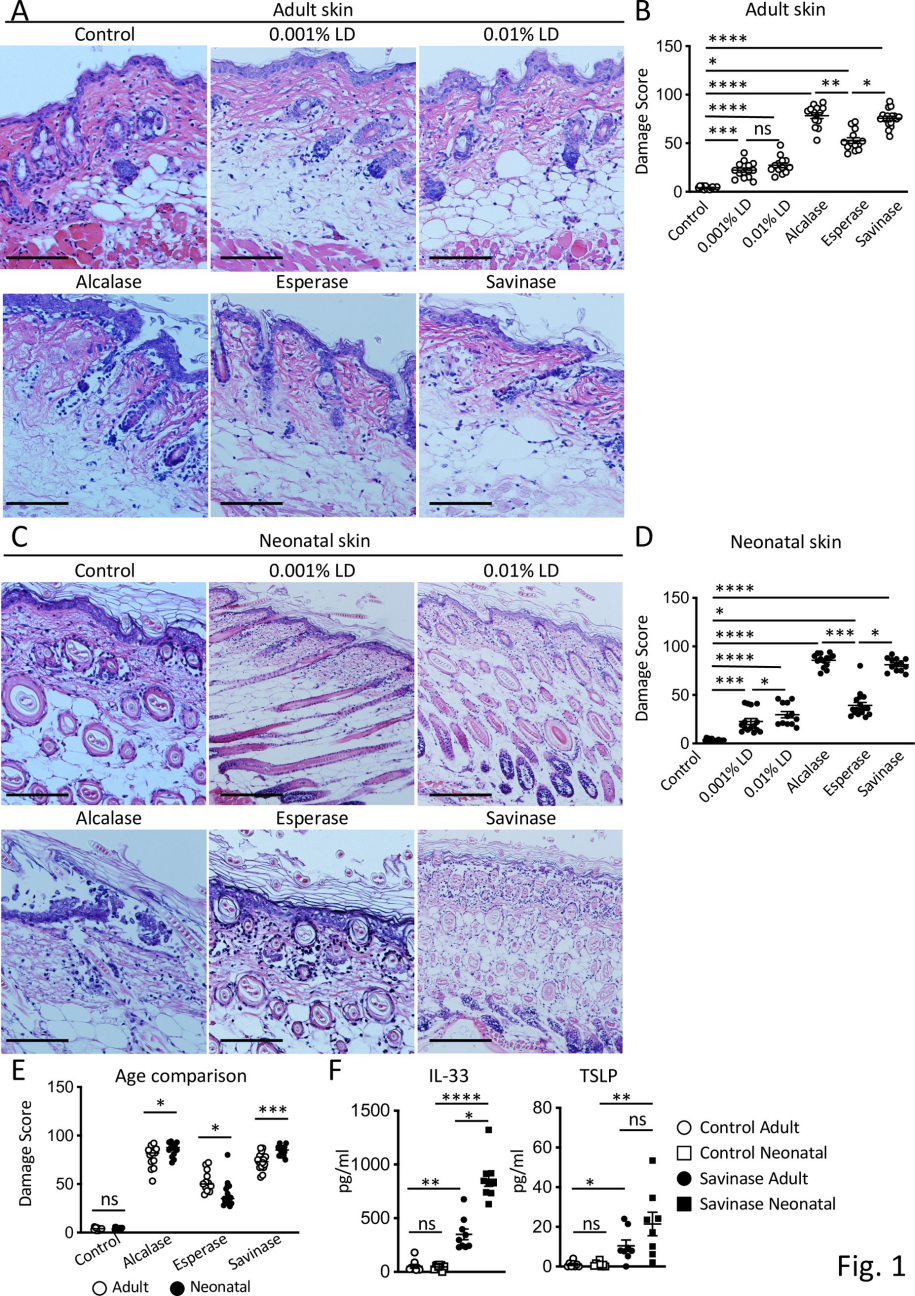
LD or microbial proteases cause skin damage in mouse skin *in vitro*. **A-E.** Representative H&E stained sections (A) and quantification of damage (B) of skin biopsies from 8-week-old adult mice. Representative H&E stained sections (C) and quantification of damage (D) of skin biopsies from 9 day-old neonatal mice, and comparison of damage between age groups (E) 24 hours after incubation with LD or proteases. Magnification, 100X. Scale bars: 100 μm. Data in E are plotted from the same experiments shown in B and D. **F.** Concentrations of IL-33 and TSLP in supernatant of adult and neonatal skin biopsies 1 hour after incubation with Savinase. Data are mean±SEMs. *p<0.05, **p<0.01, ***p<0.001, and ****p<0.001 obtained by nonparametric one-way ANOVA (B, D, F) or nonparametric Mann-Whitney U test within each exposure condition (E). ns, not significant.

Proteases in LD remove protein stains from clothes and it is possible that proteases that come in contact with the skin can break down the skin barrier [59]. To test the capacity of LD and proteases in LD to break down the skin barrier, skin biopsies were collected from the dorsal skin of 8-week-old BALB/c WT adult mice and incubated in medium containing 0.00002% Alcalase, Esperase, Savinase, or LD (0.001% or 0.1%) that contained similar percentages of proteases for 24 hours. Damage to the outer epidermis, dermis, and epidermal adnexa were assessed by histologic scoring of H&E stained skin sections. Exposure to LD resulted in epidermal thickening and increased epidermal ridges, as well as a noticeable separation of entire sections of the keratin layer from the epidermis or the epidermis from the dermis (**Fig 1A**). Adult skin biopsies incubated with 0.001% LD or 0.01% LD exhibited significantly higher damage scores (averages 22.2 and 27.3, respectively) than controls (average 4.1) (**Fig 1A and 1B**). Incubation of adult skin with 0.001% LD resulted in a trend towards lower damage score as compared to 0.01% LD (**Fig 1B**). Adult skin biopsies incubated with Alcalase, Esperase, or Savinase exhibited significantly higher damage scores than controls (**Fig 1B**), with Alcalase and Savinase resulting in higher damage scores (averages 78.4 and 75.6, respectively) than Esperase (average 52.7) (**Fig 1B**).

Since allergic disease is more prevalent in infants and food allergies usually develop in the first two years of life, we investigated the effects of LD and proteases on the neonatal skin barrier. Skin biopsies collected from the dorsal skin of 9-day-old mice were incubated with LD or proteases. As observed in adult skin, exposure to LD increased epidermal thickness and epidermal ridges while proteases caused separation of keratin layer and destruction of cells (**Fig 1C**). Neonatal skin biopsies incubated with 0.001% LD or 0.01% LD exhibited significantly higher damage scores (averages 22.7 and 29.7, respectively) than controls (average 3.6) (**Fig 1C and 1D**). LD at a concentration of 0.001% resulted in significantly lower damage scores than at 0.01% (p<0.05), indicating that LD induces damage in neonatal skin in a dose-dependent manner (**Fig 1D**). The entire epidermal matrix was destroyed in neonatal skin after exposure to proteases (**Fig 1C**) with Alcalase and Savinase causing significantly higher damage scores (averages 85.6 and 81.1, respectively) than Esperase (average 39.2) (**Fig 1D**). To further evaluate the differences in the susceptibilities of adult and neonatal skin to damage caused by LD or proteases, damage scores were compared between adult and neonatal skin within each exposure condition. Damage scores induced by Alcalase or Savinase were significantly higher in neonatal skin compared to adult skin (**Fig 1E**), indicating that neonatal skin exhibit higher susceptibility to those proteases (**Fig 1E**). In contrast, Esperase caused significantly higher damage to adult skin compared to neonatal skin (**Fig 1E**). This may be due to differences in enzyme specificity and applications among those proteases against adult versus neonatal samples.

#### Microbial proteases cause pro-atopy cytokine production

Keratinocyte-derived cytokines such as IL-33 and TSLP are released after mechanical skin injury and promote Th2 responses [49,56,60,61]. We examined the effect of skin damage induced by protease on the induction of IL-33 and TSLP. We used Savinase as it showed significant skin damage *in vitro* and is a protease commonly used in LD that is active between 15° C and 75° C [31] and does not denature until 75.5° C, which is higher than would be encountered in most washer and dryer cycles [62]. One hour after incubation of adult skin with 0.00002% Savinase, levels of IL-33 and TSLP in supernatants of skin cultures were significantly increased as compared to controls (**Fig 1F**), indicating that skin damage caused by proteases results in elevation of IL-33 and TSLP levels. Exposure of neonatal skin to Savinase resulted in significantly higher levels of IL-33 and a trend towards higher levels of TSLP than those of adult skin (**Fig 1F**), consistent with the data suggesting that neonatal skin is more susceptible to Savinase.

### Epicutaneous sensitization with LD and allergen predisposes to the development of allergic skin inflammation

**Fig 2:**
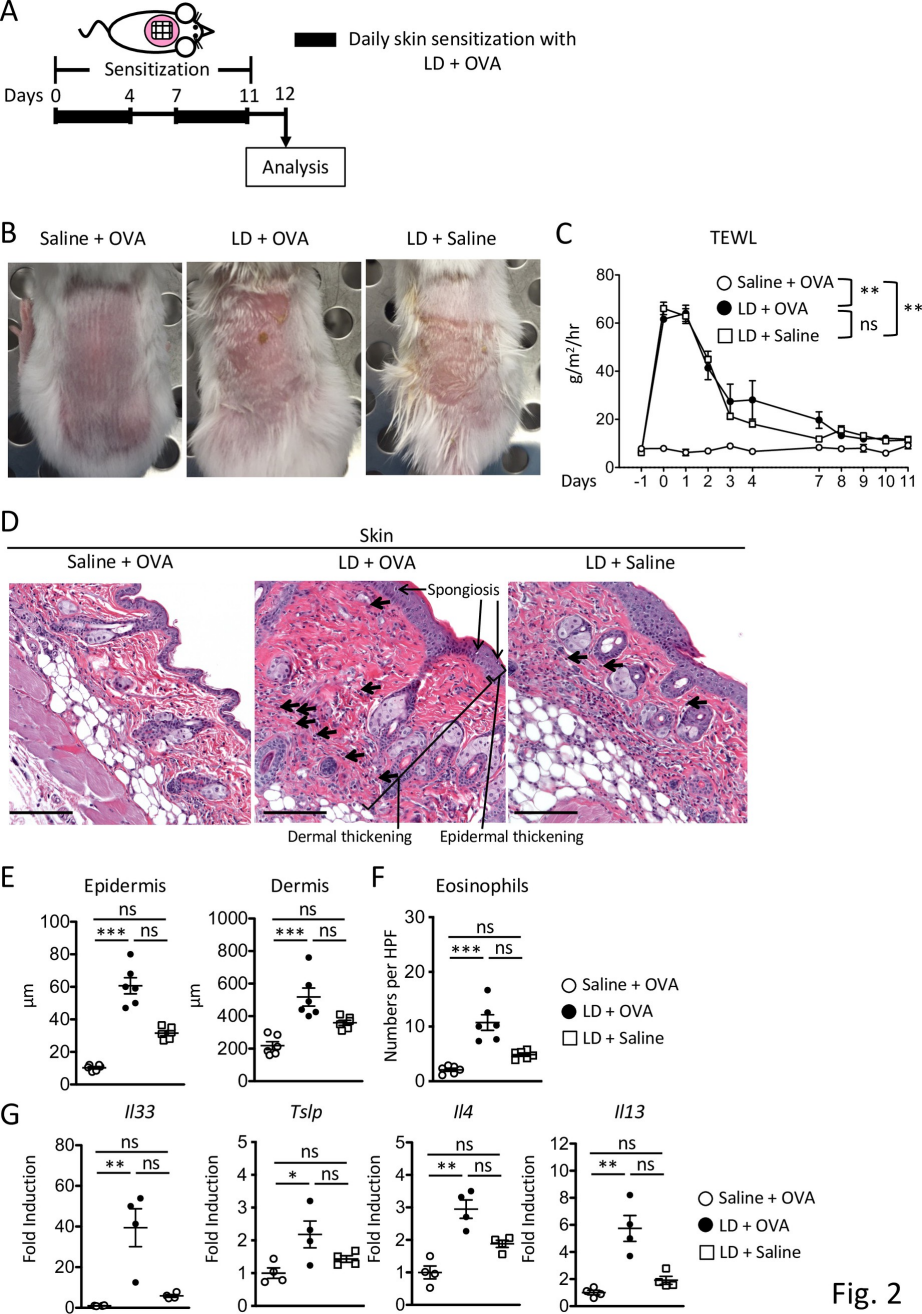
Epicutaneous sensitization with LD and allergen predisposes to the development of allergic skin inflammation. **A.** Experimental plan. **B.** Gross appearance. **C.** TEWL. **D-G.** Representative H&E stained skin sections (D), epidermal and dermal thickness (E), numbers of dermal eosinophils per HPF (F), mRNA expression of cytokines as fold induction relative to saline controls (G). Arrows in D indicate examples of eosinophils. Magnification, 100X. Scale bars: 100 μm. n = 4–5 mice per group in B-D. Data are mean±SEMs.*p<0.05, **p<0.01, and ***p<0.001 by two-way ANOVA (C) or nonparametric one-way ANOVA (E-G). ns, not significant.

Skin barrier dysfunction is a hallmark of AD, which results in dry itchy skin [12]. We hypothesized that LD causes skin damage *in vivo* that results in an enhanced allergen entry that leads to development of allergic skin inflammation. Two-to three-week-old BALB/c WT mice were topically exposed to LD or saline then OVA, or LD then saline, on unstripped skin daily over a 2-week period (**Fig 2A**). Mice exposed to LD and OVA or saline, but not saline and OVA, developed visible damage characterized by redness and scab 24 hours after the first exposure (**Fig 2B**). By day 8, after one week of daily exposure to LD, the affected area underwent gradual healing but began to scab over and remained inflamed compared to control mice exposed to saline (**S3A Fig**). Consistent with these qualitative observations, TEWL, an indicative of the epidermal permeability [63] was significantly higher in mice exposed to LD and OVA as compared to controls through day 10, indicating that the skin barrier is impaired in mice exposed to LD (**Fig 2C**). TEWL was significantly increased in mice exposed to LD and saline, suggesting that LD promotes skin barrier dysfunction in our model (**Fig 2C**). The skin barrier dysfunction in LD and OVA group was not due to daily exposure to OVA, as mice exposed to saline and OVA did not show skin lesions or an increase in TEWL (**Fig 2B and 2C**). Mice exposed to LD and OVA developed allergic skin inflammation following topical application with OVA, as evidenced by an increase in epidermal thickness, spongiosis, dermal thickness, dermal infiltration by eosinophils, and upregulated mRNA expression of *Il33*, *Tslp*, *Il4* and *Il13* as compared to mice exposed to saline (**Fig 2D–2G**), features of skin lesions in humans with AD [12]. Exposure of mice to LD and saline resulted in a non-significant trend towards Th2 responses as compared to LD and OVA group or saline and OVA (**Fig 2D–2G**). The numbers of MBP^+^ eosinophils and the frequencies of CD4^+^ T cells secreting Th2 cytokines (IL-4 and IL-13) in the skin were higher in LD + OVA group as compared to those in saline + OVA group or LD + saline group (**S3B–S3D Fig**), further supporting the induction of allergic skin inflammation in mice exposed to LD and OVA. These results indicate that skin exposure to LD results in the skin barrier dysfunction *in vivo* and the development of allergic skin inflammation following allergen exposure.

### Epicutaneous sensitization with LD and allergen predisposes to the development of esophageal eosinophilia

**Fig 3:**
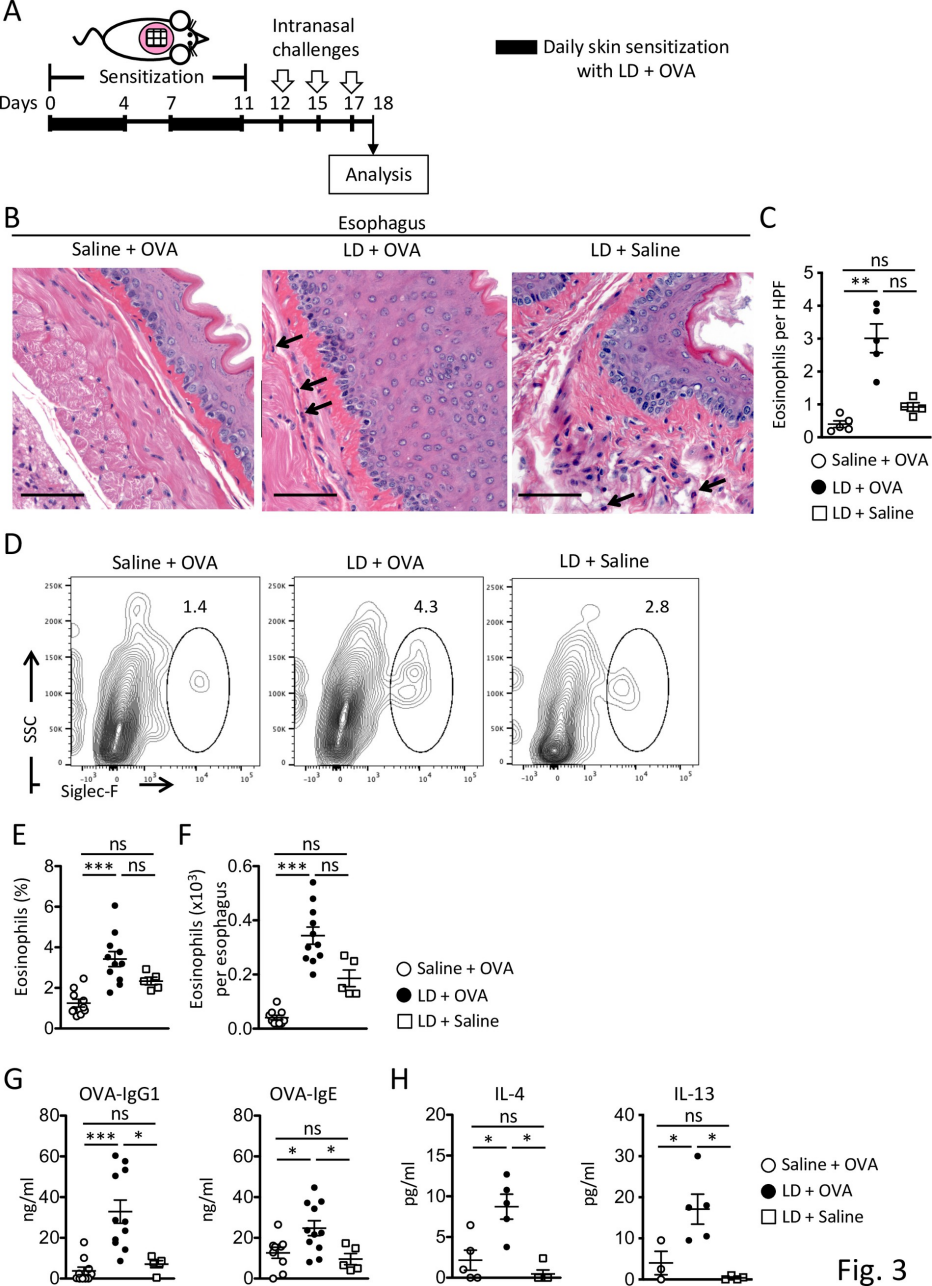
Epicutaneous sensitization with LD and allergen predisposes to the development of esophageal eosinophilia. **A.** Experimental plan. **B.** Representative H&E stained sections of the esophagus. Arrows in B indicate examples of eosinophils. Magnification, 200X. Scale bars: 50 μm. **C.** Number of eosinophils per HPF. **D-F.** Representative flow cytometry analysis (D), frequencies (E), and numbers (F) of eosinophils in the esophagus. **G.** Serum levels of OVA-specific Igs. **H.** Splenocyte cytokine secretion in response to *in vitro* OVA stimulation. Percentages of eosinophils within live, CD45^+^lin^-^ cells are shown in D. Data are mean±SEMs.*p<0.05, **p<0.01, and ***p<0.001 obtained by nonparametric one-way ANOVA (C, E-H). ns, not significant.

An abnormal skin barrier in AD [12] has been suggested as an initial entry point for food allergens [15], potentially leading to subsequent development of EoE. Given the potential role of skin sensitization in EoE, we tested the hypothesis that epicutaneous exposure to LD and allergen results in the development of eosinophilia in the mouse esophagus. LD, rather than purified proteases, was used to mimic the real-life manner of exposure to LD. Two-to three-week-old BALB/c WT mice were topically exposed to LD or saline then OVA on unstripped skin daily over a 2-week period. Intranasal challenge with OVA, which results in direct exposure of the esophagus to OVA [64], was started one day after the last cycle of skin sensitization (**Fig 3A**). Following intranasal challenge with OVA, mice exposed epicutaneously to LD and OVA exhibited higher eosinophil infiltration in the esophagus as compared to similarly challenged WT mice exposed to saline and OVA, as assessed by histology and quantified by enumeration of eosinophils (**Figs 3B and 3C and S4**). Flow cytometry analysis also demonstrated higher frequencies and numbers of eosinophils in the esophagus of LD-exposed mice compared to saline-exposed mice upon OVA challenge (**Fig 3D–3F**). Epicutaneous sensitization with LD and OVA resulted in increased generation of OVA-specific IgE and IgG1 antibodies as well as an increase in splenocyte secretion of Th2 cytokines IL-4 and IL-13 (**Fig 3G and 3H**) as compared to controls following *in vitro* stimulation with OVA, as previously reported [65,66]. These results suggest that skin LD exposure promotes development of esophageal eosinophilia and systemic sensitization following skin sensitization and intranasal challenge with allergen. Exposure of mice to LD and saline resulted in a non-significant trend towards Th2 responses as compared to LD and OVA group or OVA and saline, as indicated by an increase in levels of eosinophil infiltration in the esophagus (**Fig 3B–3F**). As expected, the LD and saline group did not exhibit allergen (OVA)-specific Th2 responses (**Fig 3G and 3H**). We have also compared the level of Th2 responses between adult and neonatal mice. Exposure of neonatal mice to LD and OVA resulted in systemic allergen sensitization, as indicated by an increase in levels of allergen-specific Th2 cytokine secretion by splenocytes. Exposure of adult mice to LD and OVA resulted in a non-significant trend towards an increase in levels of allergen-specific Th2 cytokine secretion by splenocytes, suggesting that neonatal mice are more susceptible to develop allergic responses to LD and OVA as compared to adult mice (**S2 Fig**). These results are consistent with our *in vitro* experiment indicating that neonatal skin is more susceptible to skin damage by LD at a lower concentration.

## Discussion

The skin barrier has a delicate regulatory balance to maintain its protective function that can be interrupted by chemical exposures. In this report, we examined the effects of LD exposure, which is particularly relevant to the skin barrier function and the development of allergic inflammation. We demonstrate that skin exposure to LD results in a disrupted skin barrier *in vitro* and *in vivo* mouse models, which promotes the development of experimental allergic skin inflammation and esophageal eosinophilia following allergen exposure. These results support our hypothesis that skin exposure to LD in conjunction with allergen leads to an increased risk of local and systemic allergic inflammatory responses.

Exposure of adult skin biopsies to LD or microbial proteases *in vitro* resulted in the disruption of the layers critical for the skin barrier function and the dysregulation of epidermal homeostasis, both features of human AD [12]. Proteases caused outright separation and destruction of cells, likely by cleaving adhesion proteins. Alcalase and Savinase caused more severe damages to adult skin biopsies as compared to Esperase, indicating that the distinct proteases used in LD may variably affect the skin, possibly due to their ability to function at different ranges of temperature and pH. Neonatal skin exhibited a dose-dependent disruption by LD, while adult skin demonstrated the same disruption at both doses used. In addition, the disruption of neonatal skin by proteases was more prominent than adult skin. These results indicate that neonatal skin is more susceptible to skin damage by proteases and LD, consistent with the clinical findings that infant skin is more prone to dryness and susceptible to external stimuli than adult skin due to structural differences [7,25,67]. Such skin damage was associated with elevation of IL-33 and TSLP levels in culture supernatant, consistent with the previous observations that IL-33 and TSLP are released by mechanical skin injury caused by tape stripping in mice and humans [49,56,60,61,68,69]. The production of TSLP is under genetic control and common variants in *TSLP* promote susceptibility to allergic diseases including EoE [70–72].

*In vivo* exposure of mouse skin to LD caused visible skin lesions and increased skin barrier permeability as measured by TEWL, further validating our hypothesis. Minimal consumption of LD or allergen through oral route could still occur in our model, however, these also reflect the physiological routes of exposures in infant. Whereas all the layers of the skin biopsies were exposed to LD *in vitro*, the outer keratinized barrier layer was initially exposed to LD *in vivo*, which likely resulted in enhanced LD entry to disrupt the underlying lipid membranes following skin barrier damage. The impaired response to LD in the second round of LD exposure may be due to the maturation of the skin and an increase in skin barrier function with developmental changes [73]. Whether active tolerance towards LD is induced is a future issue to be addressed. Surfactants in LD can alter the skin barrier structure, increase antigen penetration, and cause erythema, dryness, and elevated TEWL [74–80]. In line with an increase in TEWL, LD exposure followed by OVA application led to the development of allergic skin inflammation, as indicated by an increase in the levels of skin thickening, dermal eosinophils, and Th2 cytokine mRNA expression, features of human AD [79]. In addition, following intranasal challenge with OVA, mice exposed to LD and OVA via skin exhibited eosinophil accumulation in the esophagus, as seen in human EoE [81,82]. Besides the esophagus, intranasal challenge with OVA also induced eosinophilia in the lung of mice exposed to LD and OVA [56], consistent with previous reports [11,64,83].

Exposure of mice to LD and OVA resulted in systemic allergen sensitization, as indicated by an increase in the levels of allergen-specific Igs and allergen-specific Th2 cytokine secretion by splenocytes. These results are consistent with the hypothesis that skin is an important route of food sensitization [15,58]. Further, this is in line with the observations that EoE is frequently associated with AD in humans and in mice [13,14,56,84]. Previous studies found a correlation between the number and magnitude of LD doses and the likelihood of developing IgE- and/or IgG-mediated allergy [39–43]. Our data suggest that LD predisposes to skin barrier dysfunction that leads to systemic allergen sensitization and local (skin) and distal (esophagus) allergic inflammation. These findings are consistent with what was observed in workers at the LD industry, and consumers who developed multiple symptoms in the skin and the lung after exposure to proteases [33–35,37,38,42,43]. The levels of allergen-specific Th2 cytokine secreted by splenocytes from neonatal mice exposed to LD and OVA was significantly higher than those secreted by splenocytes from 8-week-old adult mice (**S2 Fig**), suggesting that neonatal mice are more susceptible to systemic allergic responses to LD and OVA than adult mice, similar to what was observed *in vitro*.

We have previously reported that allergic sensitization through tape stripped skin, which breaks the skin barrier of WT mice, induces EoE-like inflammation via the IL-33-basophil axis [56]. We have also shown that flaky tail mice that have skin barrier dysfunction due to a genetic mutation in skin barrier protein develop allergic responses to epicutaneous application of OVA alone without tape stripping, unlike WT mice [9]. Rare genetic mutations in skin barrier genes (e.g. desmoplakin and periplakin have been reported to cause EoE [85]. In this report, epicutaneous exposure to LD caused skin barrier damage without mechanical injury or a skin barrier mutation and resulted in systemic allergen sensitization. Consistently, mice exposed to saline and OVA did not show any features of allergic skin inflammation or esophageal eosinophilia, in contrast to mice exposed to LD and OVA. Skin barrier dysfunction elicited by LD likely accounts for the development of allergic inflammation, however, additional mechanisms by which LD promote these responses remain elusive. For example, LD dysregulates the skin barrier homeostasis by altering mRNA expression of lipid-metabolizing enzymes and peroxisome proliferator-activated receptors [77,78]. It is also possible that IL-33 and TSLP upregulated after skin damage caused by LD activate a network of immune cells, such as dendritic cells, basophils, and innate lymphoid cells that contribute to Th2 responses [86,87]. Further, proteases not only reduce the skin barrier function that allow entry for allergens and irritants, but also could act as adjuvants for non-proteolytic allergens such as OVA to induce allergic inflammation [59,88,89]. Interestingly, exposure of mice to LD alone resulted in a non-significant trend towards Th2 responses as compared to LD + OVA group or OVA alone, as indicated by an increase in the levels of skin thickening, Th2 cytokine mRNA expression in the skin, as well as eosinophil infiltration in the esophagus (**Figs 2D–2G and 3B–3F**). These results are consistent with a prior study indicating that the mRNA levels for IL-33 and TSLP were increased in response to LD in human bronchial epithelial cells [90], suggesting a possibility for the induction of Th2 immunity by LD, which is open to future research. These findings substantiate the recent finding that allergen sensing occurs by ripoptosome assembly resulting in intracellular maturation and release of active IL-33 [91].

While we calculated the dose as physiologically accurate as possible for the *in vitro* experiments, the concentration of LD we used for the *in vivo* experiment may be higher than the actual levels of LD residues in clothes. Indeed, *in vivo* exposure to lower concentration of LD (0.01%) failed to induce significant levels of allergen-specific responses in the LD + OVA group, as indicated by a trend towards an increase in levels of OVA-Igs (**S3E Fig**). Whether high-level exposure occurs in physiological condition is uncertain, however, the skin of human infant is thinner than adult skin [25] and infants change clothing multiple times per day in the first few months. Although the residual concentrations of LD in clothes may be low, effects of constant exposure to LD may acculmuate and significantly modulate the neonatal immune system. Human studies showed that patients with AD exhibited higher TEWL in response to cutaneous application of 0.5%-1% of pure sodium lauryl sulfate (SLS) [92,93]. Mouse studies used 4% of SLS alone to break the skin barrier in NC/Nga mice, a spontaneous mouse model of eczema [94,95]. In this study, mice were exposed to LD according to previous studies suggesting that exposure to LD increased antibody response compared to enzyme alone, suggesting that other LD ingredients stimulated the allergic response [41,96,97]. Notably, WT mice that are not prone to atopy unless sensitized were employed. On all accounts, the present data support that LD plays a role in promoting allergen sensitization through barrier-impaired skin. Following this proof-of-concept study, a future direction could be to invest a more extended experimental model *in vivo* using long-term low-level exposure, which is more similar to real-life exposures.

In summary, these results suggest that LD may be an important environmental factor that impairs the skin barrier and leads to allergen sensitization in early life, and therefore have been a contributory factor the allergy epidemic. Our study is in line with numerous studies that repeatedly supported the importance of skin allergen sensitization in food allergy [98] as well as the association between atopic diseases, including atopic dermatitis, food allergy, and allergic asthma. Supportedly, there is a close relationships between EoE and atopic dermatitis based on transcriptomic analysis [99]. Also, others have shown that EoE is part of the atopic march associated with atopic dermatitis [100]. It is notable that currently used LD contain a number of ingredients that may promote impaired barrier function and release of pro-atopy cytokines. LD possess disruptive effects on the barrier function of human bronchial epithelial cells [90], suggesting inhalation as a different route of LD exposure. Our findings are in line with the ‘hygiene hypothesis’ that invokes the role of improved hygiene as an environmental factor contributing to the increase in the prevalence of allergies. Given low-levels of exposure to LD residue on clothing, it might take years for adults to develop allergic sensitization, and this could vary depending on genetic predisposition, skin condition, and the level of the exposure to LD residue based on their clothes-washing habits. However, LD residue on clothing could be particularly hazardous to newborns, whose pH is already high, ranging from 6.3 to 7.5 [101], the skin barrier more fragile, and whose immature immune system is more vulnerable to dysregulation from exposure to microbial protases and other LD ingredients. Indeed, very early onset of EoE in the first year of life is a common presentation [102]. A prophylactic daily moisturization until 6 months of age prevents AD in high-risk newborns, suggesting that skin hydration in this critical time may have a protective effect [103]. We used mouse models to study the role of LD on the development of allergic responses to specific allergens under controlled environmental conditions within defined genetic backgrounds at specific timing, which is not possible in human subjects. While the clinical significance of our findings using the skin biopsies exposed to LD *in vitro* needs to be established by examining the effects of LD on human infant skin, *in vivo* experiments established that skin exposure to LD results in the skin barrier dysfunction *in vivo* and the development of allergic skin inflammation and esophageal eosinophilia following allergen exposure. The study presented herein provides initial support for the hypothesis that LD (whether from laundry or other sources) may be involved in esophageal eosinophilia. These collective findings support the hypothesis that LD may be an important environmental factor that impairs the skin barrier and leads to allergen sensitization in early life and contribute to esophageal eosinophilia and other allergic diseases.

In conclusion, this work has important implications for the development of new preventive strategies since moisturization of the skin at any stage of life, especially infant, as well as the choice of type and/or dose of LD in early life to protect skin barrier might be an effective target for the prevention of allergies.

## Supporting information

S1 Fig Examples of skin damage evaluation *in vitro*. (TIF)

S2 Fig Secretion of IL-4 and IL-13 by splenocytes from adult and neonatal mice. Data are mean±SEMs. *p<0.05 obtained by nonparametric one-way ANOVA. ns, not significant. Data of neonatal mice are shared with Fig 3H.
(TIF)

S3 Fig Epicutaneous sensitization with 10%, but not 0.01%, LD and allergen predisposes to the development of allergic skin inflammation. **A.** Gross appearance after one week of daily exposure to 10% LD. **B-D.** Representative MBP stained skin sections (B), numbers of MBP^+^ eosinophils per HPF (C), the frequencies of CD4^+^ T cells secreting Th2 cytokines in the skin (D) of mice exposed to 10% LD. **E.** Serum levels of OVA-specific Igs in mice exposed to 0.01% LD. Arrows in B indicate examples of eosinophils. Magnification, 100X. Scale bars: 100 μm. n = 5 mice per group. Data are mean ± SEMs. ***p<0.001 obtained by nonparametric one-way ANOVA (C, D) or nonparametric Mann-Whitney U test (E). ns, not significant.
(TIF)

S4 Fig Epicutaneous sensitization with LD and allergen predisposes to the development of esophageal eosinophilia. **A, B.** Representative MBP stained sections of the esophagus (A) and numbers of MBP^+^ eosinophils per HPF (B). Arrows in A indicate examples of eosinophils. Magnification, 100X. Scale bars: 100 μm. n = 5 mice per group in A, B. Data are mean ± SEMs.***p<0.001 obtained by nonparametric one-way ANOVA (B). ns, not significant.
(TIF)

S1 Dataset (XLSX)
