# Supplementary figures and images for: Laundry detergent promotes allergic skin inflammation and esophageal eosinophilia in mice
Source: PLoS One. 2022 Jun 27;17(6):e0268651. doi: 10.1371/journal.pone.0268651 (PMC9236249; doi:10.1371/journal.pone.0268651)

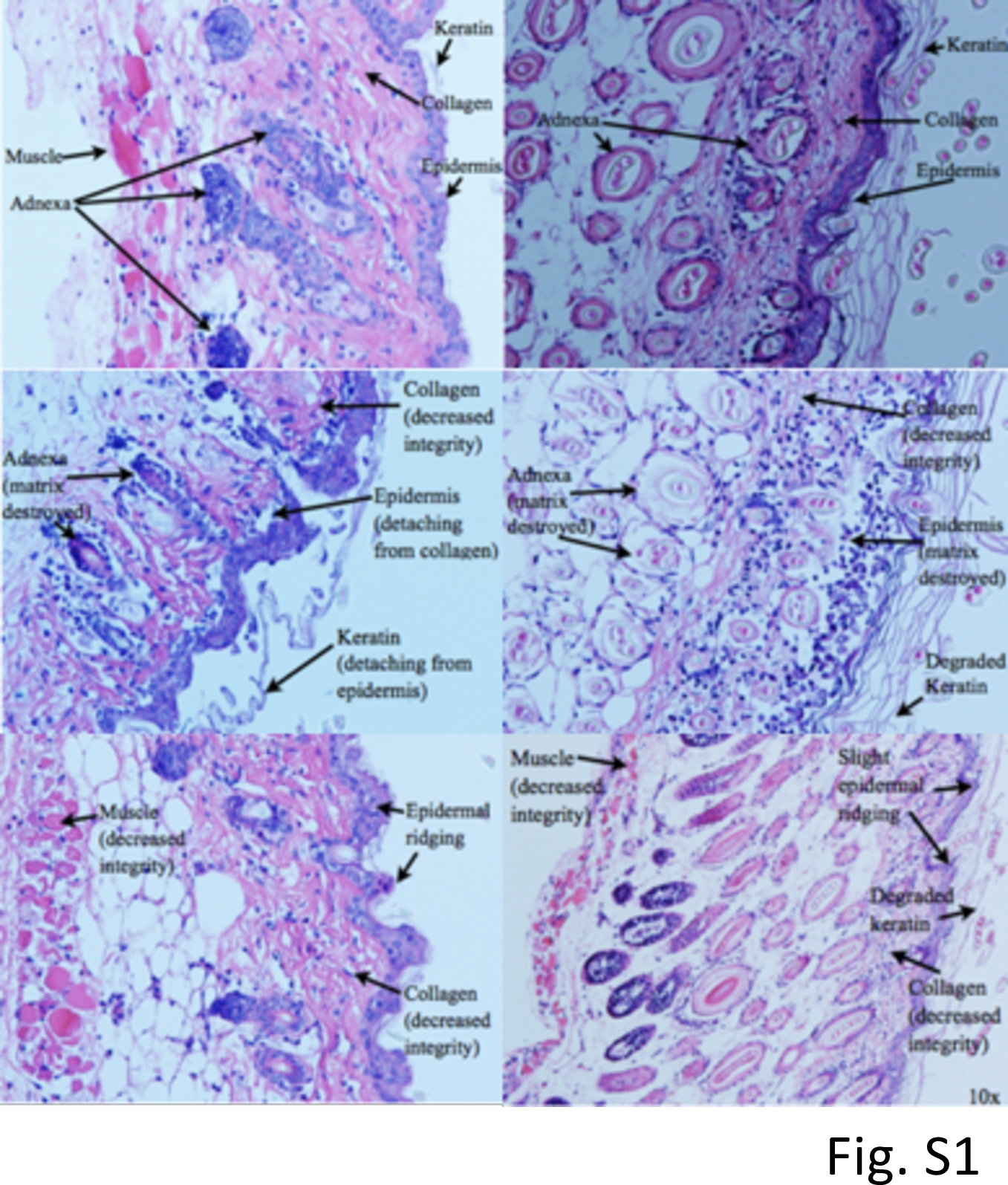

Supplement: S1 Fig — (TIF) [file pone.0268651.s001.tif]

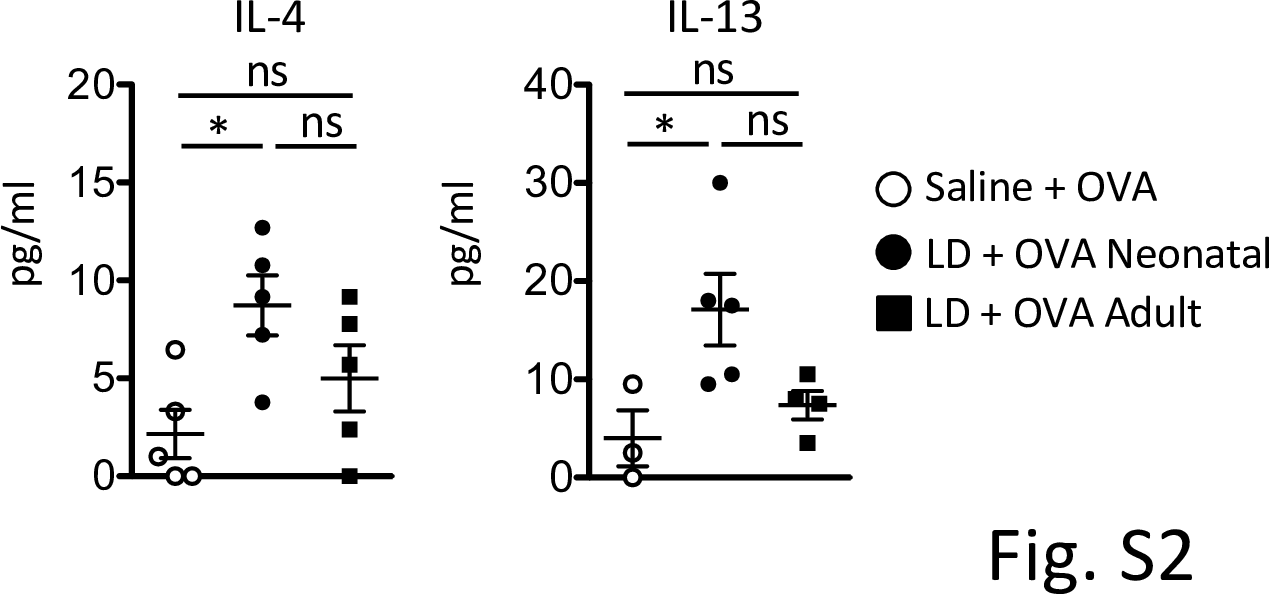

Supplement: S2 Fig — Data are mean±SEMs. *p<0.05 obtained by nonparametric one-way ANOVA. ns, not significant. Data of neonatal mice are shared with Fig 3H. (TIF) [file pone.0268651.s002.tif]

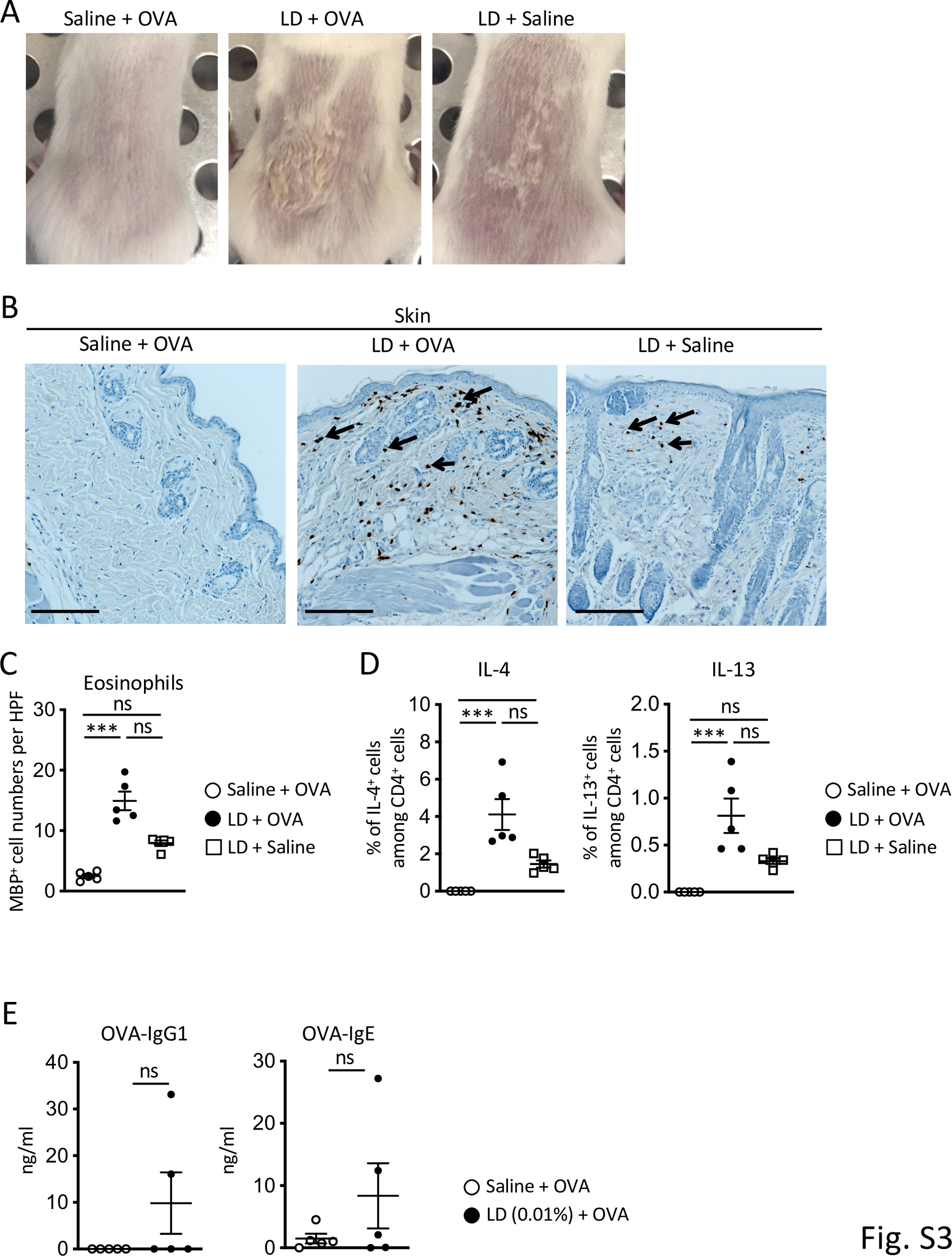

Supplement: S3 Fig — A. Gross appearance after one week of daily exposure to 10% LD. B-D. Representative MBP stained skin sections (B), numbers of MBP+ eosinophils per HPF (C), the frequencies of CD4+ T cells secreting Th2 cytokines in the skin (D) of mice exposed to 10% LD. E. Serum levels of OVA-specific Igs in mice exposed to 0.01% LD. Arrows in B indicate examples of eosinophils. Magnification, 100X. Scale bars: 100 μm. n = 5 mice per group. Data are mean ± SEMs. ***p<0.001 obtained by nonparametric one-way ANOVA (C, D) or nonparametric Mann-Whitney U test (E). ns, not significant. (TIF) [file pone.0268651.s003.tif]

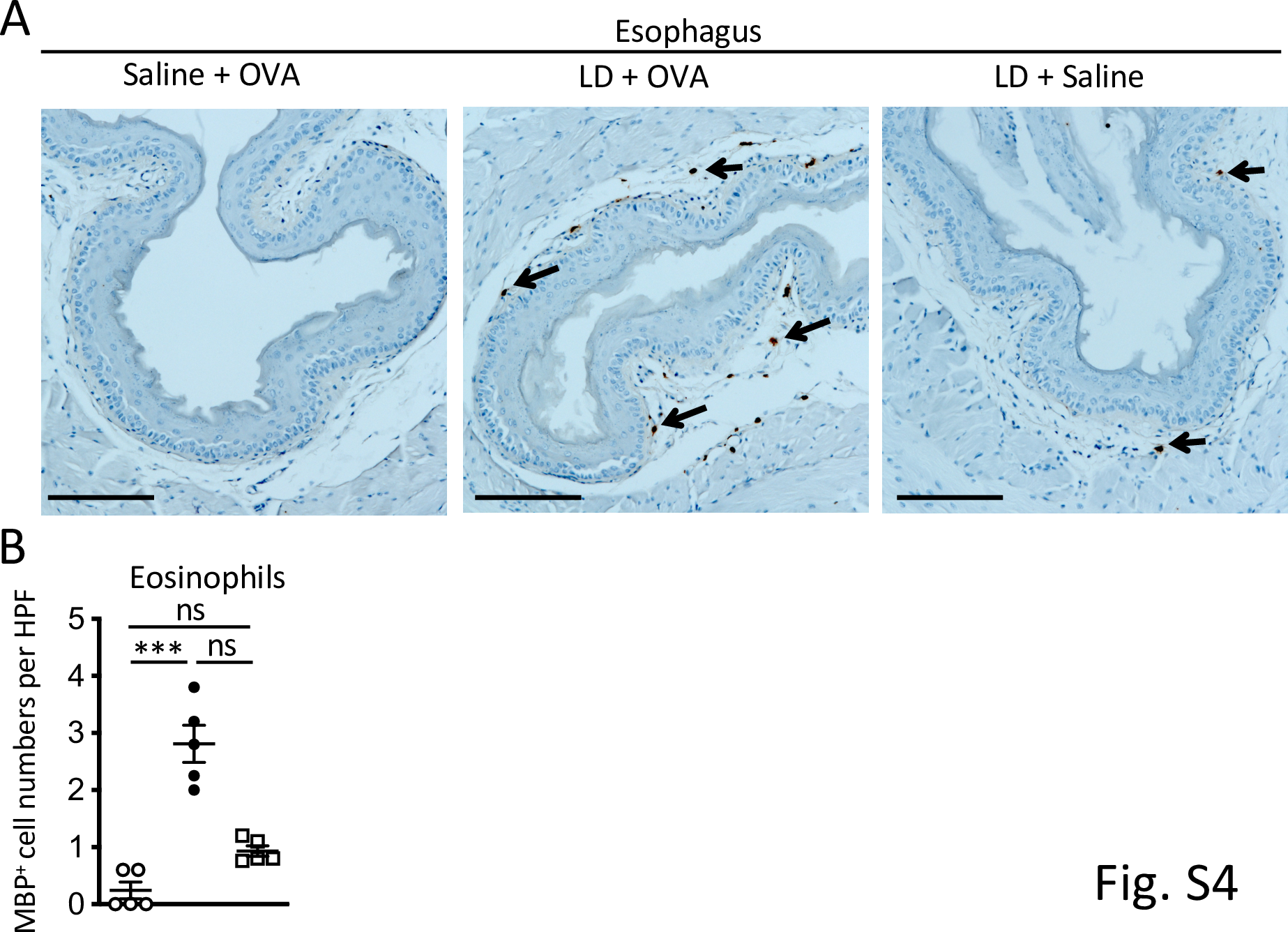

Supplement: S4 Fig — A, B. Representative MBP stained sections of the esophagus (A) and numbers of MBP+ eosinophils per HPF (B). Arrows in A indicate examples of eosinophils. Magnification, 100X. Scale bars: 100 μm. n = 5 mice per group in A, B. Data are mean ± SEMs.***p<0.001 obtained by nonparametric one-way ANOVA (B). ns, not significant. (TIF) [file pone.0268651.s004.tif]
